# Current Practice of Surgery for Benign Goitre—An Analysis of the Prospective DGAV StuDoQ|Thyroid Registry

**DOI:** 10.3390/jcm8040477

**Published:** 2019-04-08

**Authors:** Detlef K. Bartsch, Cornelia Dotzenrath, Christian Vorländer, Andreas Zielke, Theresia Weber, Heinz J. Buhr, Carsten Klinger, Kerstin Lorenz, the StuDoQ/Thyroid Study Group

**Affiliations:** 1 Department of Visceral-, Thoracic- and Vascular Surgery, Philipps-University Marburg, 35043 Marburg, Germany; 2 Department of Endocrine Surgery, Helios University Hospital Wuppertal, 42283 Wuppertal, Germany; cornelia.dotzenrath@helios-kliniken.de; 3 Department General and Endocrine Surgery, Bürgerhospital Frankfurt/Main, 60318 Frankfurt, Germany; c.vorlaender@buergerhospital-ffm.de; 4 Department of Endocrine Surgery, Diakonie-Klinikum Stuttgart, 70176 Stuttgart, Germany; andreas.zielke@diak-stuttgart.de; 5 Department of Endocrine Surgery, Katholisches Klinikum Mainz, 55131 Mainz, Germany; t-weber@kkmainz.de; 6 German Society of General and Visceral Surgery (DGAV), 10117 Berlin, Germany; hbuhr@dgav.de (H.J.B.); carsten.klinger@dgav.de (C.K.); 7 Department of Visceral-, Vascular and Endocrine Surgery, University Medical Centre Halle, 06120 Halle, Germany; kerstin.lorenz@uk-halle.de

**Keywords:** indication, thyroid resection, registry, recurrent laryngeal nerve palsy, hypoparathyroidism

## Abstract

Background: To evaluate the current indications, resection strategies and short-term outcomes of surgery for benign goitre in a country with endemic goitre. Methods: Data of patients who underwent surgery for benign goitre were retrieved from the prospective StuDoQ/Thyroid registry and retrospectively analysed regarding the patient’s demographics, indications for surgery, surgical procedures, histology, and perioperative outcomes. Results: In a 15-month period, 12,888 patients from 83 departments underwent thyroid resections for benign conditions. Main indications for surgery were exclusion of malignancy (68%), compression symptoms (20.7%) and hyperthyroidism (9.7%). Preoperative fine needle aspiration cytology was performed in only 12.2% of patients with the indication “exclusion of malignancy”. Thyroidectomy (49.8%) or hemithyroidectomy (36.9%) were performed in 86.7% of patients. Minimally invasive or alternative surgical techniques were applied in only 2.2%. Intraoperative neuromonitoring was used in 98.4% of procedures, in 97.5% of patients at least one parathyroid gland was visualized, and in 15.3% of patients parathyroid tissue was autografted, respectively. The rates of unilateral and bilateral transient recurrent nerve palsy were 3.6% and 0.07% of nerves at risk, the rate of transitory hypoparathyroidism was 15.3%. The rates of postoperative bleeding and wound infections requiring reoperation were 1.4% and 0.07%, respectively. Conclusions: The indication “exclusion of malignancy” is made too liberally, and there is a strong attitude to perform complete thyroid resections. Postoperative hypoparathyroidism is the major complication after surgery for benign thyroid disease, thus requiring more awareness.

## 1. Introduction

Germany is an endemic goitre area and thyroid resections are among the most frequent visceral operations, with approx. 70,000 procedures being carried out per year [1,2]. Thyroid resections are performed by general surgeons, as well as specialized endocrine surgeons in hospitals of all care levels. National and international guidelines for thyroid surgery, including quality criteria, have been developed on the basis of retrospective cohort studies of specialized centres [3,4,5,6,7], since prospective randomized-controlled trails are largely missing. The German Society for General and Visceral Surgery (DGAV) established the StuDoQ/Thyroid registry in close cooperation with the German Society of Endocrine Surgeons (CAEK) for quality control in thyroid surgery to close the gap between the somewhat virtual world of prospective randomized trials, retrospective cohort studies of tertiary referral centres, and daily practice. Health data of insured persons are increasingly evaluated by health insurances to evaluate the quality and practice of thyroid surgery and to make it transparent to the public [8,9]. It has been shown by a retrospective analysis of insurance data that only 9% of patients with thyroid resections underwent preoperative calcitonin screening [8], suggesting that actual guideline recommendations [3,4,5,6,7] are not fully implemented in daily practice. It has also been shown that the resection strategy for benign goitre changed from subtotal resections to thyroidectomy in Germany between 2005 to 2011 [10]. However, recently, some experts have again made a plea for individualizing the extent of resection for endemic goitre instead of heading for routine total thyroidectomy [11].

Aim of the present study was to evaluate the current practice of thyroid surgery for benign goitre in Germany based on the StuDoQ/Thyroid registry, including the indication for operation, operative strategies and perioperative outcomes.

## 2. Materials and Methods

The StuDoQ/Thyroid Registry of the DGAV was designed as a prospective, multicentre database with web-based data entry. The register is managed by the DGAV and was developed in close cooperation with the board of the German Society of Endocrine Surgeons (CAEK). Relevant outcome and quality indicators were selected on the basis of the German S2k guidelines [3,4,5] for the surgical treatment of benign and malignant goitre, the certification guidelines for competence centres for thyroid/parathyroid surgery of the DGAV (www.dgav.de), and the EUROCRINE registry (https://eurocrine.eu). 52 parameters were defined by a browser-based electronic case report form (eCRF). The registry was prospectively maintained since April 2017. Data from participating centres were entered in pseudonymized form at the institutional level without an over-institutional identifier in the prospective StuDoQ/Thyroid registry. The data were subject to an immediate automatic plausibility check with automatic error indication, which avoids many typical and predictable input errors.

All participating institutions have to approve the correct number of thyroid resections entered in the registry by a written confirmation of the hospitals’ controller. In addition, the completeness and correctness of the data will be checked as part of the regular certification audits for the certified competence and reference thyroid centres of the DGAV.

All enrolled patients signed an informed consent for data collection, and approval for data management was obtained from the TMF (Technologie und Methodenplattform für die vernetzte medizinische Forschung).

For the present study, all patients who underwent thyroid resections for benign pathologies between 1 April 2017 and 6 July 2018 were extracted from the StuDoQ/Thyroid registry. Data were extracted in anonymised form and retrospectively analysed with respect to demographics, indication for surgery, types of surgical procedures, histology, and perioperative outcomes.

The fine needle aspiration cytology specimens were classified according to the Bethesda criteria [12]. The postoperative function of the recurrent laryngeal nerve was determined by laryngoscopy within the first 5 postoperative days by an independent head and neck specialist. Vocal cord immobility was defined as palsy, impaired, but notable vocal cord movement was defined as incomplete palsy. Both conditions were documented as dysfunction of the recurrent laryngeal nerve. Pre-existing recurrent laryngeal nerve palsies were excluded from the present analysis. The palsy rate was calculated per nerves at risk (NAR). The nerve dysfunction was defined as transient if it completely recovered within 6 months, and permanent if it persisted after 6 months. Postoperative hypoparathyroidism was defined as a serum level of intact parathormone <15 pg/L and serum calcium <2.1 mmol/L with the necessity of calcium substitution. The postoperative hypoparathyroidism was defined as transient, if it completely recovered within 6 months, after that time it was considered permanent.

### Statistical Analysis

Continuous variables are presented as means (standard deviation) and categorical variables as proportions. Quantitative variables were compared using the Student’s *t*-test and qualitative variables (e.g., gender) using the chi-square test or Fisher’s exact test as appropriate. All reported probability values (*p*-values) are based on two sided tests; the level of statistical significance was set at *p* < 0.05. Analyses were performed using SPSS 23.0 (IBM, Chicago, IL, USA, 2017).

## 3. Results

A total of 17,702 patients from 83 surgical departments were entered into the StuDoQ/Thyroid Registry in the first 15 months. The data of 12,888 patients with benign goitre, who were discharged before 6 July 2018, were analysed. All levels of thyroid surgery care were represented, since 34% (*n* = 29) of participating clinics operated on fewer than 50 patients, while 18% (*n* = 15) of clinics operated on more than 300 patients per year (Table 1). Almost two thirds of patients (62.9%, *n* = 8102) were operated on in clinics that performed more than 300 procedures per year (Table 1).

74.1% (*n* = 9550) women and 25.9% (*n* = 3338) men with a median age of 55 (range 12–88) years underwent thyroid surgery. Only 61 (0.47%) patients were younger than 18 years, and 1556 (12.1%) patients were older than 70 years. 10.3% (*n* = 1328) of patients (1048 women, 280 men) were overweight with a body mass index >35. A total of 13.3% (*n* = 1714) patients had an increased perioperative risk, 13% (*n* = 1671) of patients were classified as ASA3, and 0.3% (*n* = 43) patients as ASA4.

The main indication for surgery was exclusion of malignancy in 68% (*n* = 8762) of patients, followed by compression symptoms in 20.7% (*n* = 2666) patients and hyperthyroidism in 9.7% (*n* = 1248) patients (Table 2). 95.8% (*n* = 12,347) of patients underwent initial surgery and 4.2% (*n* = 541) patients had cervical reoperations on the affected side(s).

Preoperative calcitonin screening was performed in 62.6% (*n* = 8068) of patients. A preoperative fine needle aspiration cytology (FNAC) was performed in only 12.2% (*n* = 1068) of the 8762 patients with the main surgical indication “exclusion of malignancy”. In 60 (5.6%) of these patients, the FNAC result was cancer (Bethesda VI) or suspicion of malignancy (Bethesda V). In 22.9% (*n* = 245) of these patients a follicular neoplasia (Bethesda IV), in 22,1% (*n* = 236) atypia of unclear significance or a follicular lesion of unclear significance (Bethesda III) and in 32,9% (*n* = 352) of these patients a benign finding was diagnosed, whereas in 15.2% (*n* = 162) of these patients, the FNA was not diagnostic, respectively.

Preoperative laryngoscopy for the assessment of vocal cord function was performed in 97.6% (*n* = 12,578) patients and normal results were obtained in 96.7% (*n* = 12,462) of patients. 0.9% (*n* = 116) patients had a unilateral palsy of the recurrent laryngeal nerve (RLNP), and 0.04% (*n* = 4) patients had a bilateral RLNP.

The most frequently performed thyroid resections were either thyroidectomy (49,8%, *n* = 6386) or hemithyroidectomy (36.9%, *n* = 4768), accounting for more than 85% of operations (Table 3). The proportion of thyroidectomies was the highest in the case of Graves’ disease, with 92.1%.

In 97.8% (*n* = 12,602) of patients, conventional open surgery was performed, with minimally invasive video-assisted thyroidectomies (MIVAT) being performed in 2.2% (*n* = 286) of patients. Robotic-assisted thyroidectomies or resections via a combined axillary breast access (ABBA) were an absolute exception, with 14 and 3 (0.01%) procedures, respectively. Only 0.18% (*n* = 24) of patients required a sternotomy for the resection of benign thyroid disease. The median operative time for hemithyroidectomy (75, range 22–380 min) and thyroidectomy (109, range 42–384 min) were similar compared to unilateral (63, range 18–265 min) and bilateral subtotal resections (100, range 28–246 min, *p* > 0.05).

Visualisation of the recurrent laryngeal nerve (RLN)—as recommended in the guidelines—was performed in 99.8% (*n* = 12,874) of patients. Intraoperative neuromonitoring (IONM) of the RLN was used in 98.4% (*n* = 12,686) of patients, whereby intermittent IONM (82.6%) was significantly more used than continuous IONM (17.4%, *p* = 0.02, Table 4). IONM of the superior laryngeal nerve, however, was only performed in 7.7% (*n* = 997) of patients (Table 4). In 97.5% (*n* = 12,564) of patients, at least one parathyroid gland was identified intraoperatively, with no significant differences between the entities (Table 4). Autotransplantation of parathyroid tissue was undertaken in 15.3% (*n* = 1875) of patients, most frequently after resections for Graves‘ disease, with 22.3% (Table 4).

The median weight of resected nodular goitre specimens was 48 grams; 10.1% (*n* = 916) weighted less than 10 grams, and 9.6% (*n* = 874) more than 100 grams. Histopathological examination revealed the following main diagnoses: nodular goitre in 70.4% (*n* = 9069) of patients, follicular adenoma in 14.1% (*n* = 1817), Graves’ disease in 7.2% (*n* = 925) and other pathologies in 8.3% (*n* = 1077) of patients, respectively.

Postoperative laryngoscopy was performed in 95.6% (*n* = 12,320) of patients. This revealed a dysfunction in 672 of 18793 (3.6%) nerves at risk (NAR), with unilateral dysfunction in 658 (3.5%) patients and bilateral dysfunction in 14 (0.07%) patients (Table 5). The rate of RLNP was highest (3.9%) after surgery for Graves’ disease. At the evaluation date, 525 of the 672 NAR with early postoperative dysfunction should have had the 6-months follow-up laryngoscopy. This result was documented for 244 (47%) NAR, and 119 (48.8%) of examined nerves showed a permanent palsy. Based on these results, one can extrapolate a rate of permanent RLNP of about 1.4% per NAR.

A postoperative hypocalcemia with the necessity of calcium substitution was present at discharge in 15.3% (*n* = 1965) of patients. The rate of postoperative transient hypoparathyroidism was significantly higher in patients with Graves’ disease (28.4%) than in patients with multinodular goitre (15%) and other entities (11,9%, *p* < 0.05, Table 5). A 6-month follow-up examination was documented for 1021 (51.9%) patients at the evaluation date, 132 (12.9%) of those showed a permanent postoperative hypoparathyroidism. Based on these data, a rate of permanent hypoparathyroidism of 1.5% to 3.5% depending on the operated entity can be calculated.

Reoperations for postoperative bleeding or wound infection had to be performed in 1.4% (*n* = 180) and 0.07% (*n* = 11) of patients (Table 5).

## 4. Discussion

In 2013, approximately 72,000 thyroid surgeries were performed by 852 departments in Germany [9]. It can therefore be assumed that almost 10% of departments which perform thyroid resections in Germany entered their data in the StuDoQ/Thyroid registry and that about 15% of patients undergoing thyroid resections during the 15-months evaluation period were recorded. Although this rate cannot be regarded as representative, since only a coverage rate of more than 20% is considered representative [13], clear trends with regard to indications, surgical standards and outcome quality of thyroid surgery can be derived from these prospective registry data.

The presented data clearly show a relatively radical attitude towards the extent of resection in benign thyroid pathologies, since more than three quarters of the operations were thyroidectomies or hemithyroidectomies. Partial thyroid resections, especially nodule-oriented partial resections, play only a minor role. Already between 2005 and 2011, the rate of total thyroidectomies and hemithyroidectomies had increased from 37% to 73%, while the rate for partial or subtotal resections decreased by about 60% [10]. This is obviously an ongoing worldwide tendency, although recently some experts have made a plea for individualizing the extent of resection in endemic goitre instead of heading for routine thyroidectomy [11].

The presented data also show that minimally invasive thyroid resections or alternative approaches such as robotic-assisted thyroidectomy currently play only a marginal role in Germany with about 2% of all interventions. This is in contrast to the impressions promoted by some media and the relatively high number of reports on alternative approaches for thyroid resections in recent years.

It is often critically noted that, especially in Germany, thyroid resections are indicated too liberally for so-called “cold”, potentially malignant nodules, since the preoperative tools recommended in the guidelines for determining the risk of malignancy are not completely used [2,14]. In the present study, the main indication for surgery was “exclusion of malignancy” in two thirds of patients, but a preoperative FNAC was performed in only 12%, and a preoperative calcitonin screening in only 60% of these patients. Thus, current guideline recommendations [3,4,5,6,7] are not well implemented across the country. This underscores a previous retrospective insurance data analysis of 25,600 patients with nodular goitre [8]. This study demonstrated that more than a quarter of the operations (approx. 20,000) in Germany were performed because of “suspicious” thyroid nodules, but the preoperative calcitonin screening and a FNAC were performed in only 9% and 21% of patients, respectively [8]. Since the indication for surgery in potentially suspicious thyroid nodules is also significantly triggered by the patient, further efforts should be undertaken to educate patients, despite a more consistent use of the diagnostic options by the physicians.

The operative standards in participating clinics of the registry were high. The RLN was visualised in almost every operation, and IONM was used in more than 98% of procedures for the potential protection of the RLN, although IONM is not recommended in the guidelines [3,4,5,6,7]. This result confirms the data from a questionnaire survey conducted in 2012, where IONM was already used in 91% of thyroidectomies in Germany [15]. However, continuous IONM was used in only 17.4%, less than expected, although it might be superior to the intermittent IONM with regard to the real-time detection of traction injury of the RLN [16]. In more than 98% of all interventions, at least one parathyroid gland was identified and an autotransplantation of devascularized parathyroids was routinely performed as required by the guidelines [3,4,5,6,7].

The StuDoQ/Thyroid registry also seems to realistically reflect the complication rates. The rate of transient RLNP is 3.6%, with reference to NAR, and is thus somewhat higher than in previous European registry studies, with 2.9% and 3.5% [17,18], but lower than the 6.9% rate in a German prospective randomized multicentre study CLIVIT [19] conducted at 13 institutions. However, only 14 (0.07%) patients experienced a bilateral RLNP, which is in the lower range of the more recent literature [19,20,21,22,23]. In 51% of the patients who were re-examined after 6 months, the RLNP had completely recovered. Thus, one can extrapolate a rate of permanent RLNP of 1.4% for the total cohort, which is in the middle range of the RNLP rates (0.9–2.9%) reported in prospective randomized trials [10,19,21,22,23].

The rate of transient postoperative hypoparathyroidism at discharge was comparatively high, with 15.3% in the total collective [10,19,20,21,22,23], whereby the rate was highest after surgery for Graves’ disease. Independent of the entity, at least 80% of patients who had already had a 6-months follow-up examination showed a complete recovery of the parathyroid function. Thus, the rate of permanent postoperative hypoparathyroidism ranges from 1.5% to 3.5%, depending on the entity. These rates are in the upper range of the recent literature [10,19,20,21,22,23]. Since permanent postoperative hypoparathyroidism is a debilitating complication for the affected patients, even more attention should be paid in the future to avoid this complication. The rates of postoperative bleeding and wound infections requiring revision was comparatively low in the overall collective with 1.4% and 0.07%, respectively [10,19,20,21,22,23].

The present study has some drawbacks and some strengths. Although the data are prospectively documented in the StuDoQ/Thyroid registry and complete recording of all consecutive cases must be confirmed by the respective controlling of the participating centres every year, the current analysis has all of the limitations of a retrospective study. The 6-months follow-up data regarding RLNP and postoperative hypoparathyroidism were not available for about half of the patients. However, the present study based on the StuDoQ/Thyroid registry gives a clear picture of the actual trends of thyroid surgery for benign goitre in Germany, since almost 13,000 patients were documented in the previous 15 months.

In summary, the indication “exclusion of malignancy” or the so-called “diagnostic” (hemi)thyroidectomy can be made less liberally, if the recommended armamentarium of preoperative diagnostic options were to be fully used. Currently, thyroid resections are mostly hemi- or thyroidectomies, whereas partial resections play only a very minor role. The intraoperative standards to avoid injury of the RLN and the parathyroids are high, although postoperative hypoparathyroidism is still a substantial problem which should be focused on.

## Figures and Tables

**Table 1:** Number of thyroid procedures and operated entities of participating clinics.

Number of Cases Per Clinic (Clinics/%)	Multinodular Goitre (*n* = 9069)	Graves’ Disease (*n* = 925)	Other Entities * (*n* = 2894)	All Entities (*n* = 12,888)
2–50 (29/34%)	255 (2.8%)	17 (1.8%)	105 (3.6%)	377 (2.9%)
51–100 (17/20%)	639 (7%)	66 (7.1%)	238 (8.2%)	943 (7.3%)
101–300 (24/28%)	2335 (25.7%)	272 (29.4%)	859 (29.7%)	3466 (26.9%)
>300 (15/18%)	5840 (64.4%)	570 (61.6%)	1692 (58.4%)	8102 (62.9%)
total (83/100%)	9069 (100%)	925 (100%)	2894 (100%)	12,888 (100%)

* including follicular adenoma, autonomous adenoma, regressive thyroid nodule, all forms of thyroiditis, c-cell hyperplasia and normal thyroid.

**Table 2:** Main indication for thyroid surgery in histologically benign lesions.

Main Indication for Surgery	No. of Patients	%
Exclusion of malignancy	8762	68.0
Compression symptoms	2666	20.7
Hyperthyroidism	1248	9.7
Hyperparathyroidism	154	1.2
Redo surgery for benign finding	58	0.4
Total	12,888	100

**Table 3:** Extent of thyroid resections according to entity.

Type of Surgery	All Entities	Multinodular Goitre	Graves’ Disease	Other Entities *
Thyroidectomy	6386 (49.8%)	4547 (50.2%)	852 (92.1%) #	987 (34.1%)
Hemithyroidectomy	4768 (36.9%)	3376 (37.2%)	47 (5.1%)	1345 (46.4%)
Hemithyroidectomy, contralateral node excision	469 (3.6%)	370 (4.1%)	2 (0.2%)	97 (3.4%)
Hemithyroidectomy, contra-lateral subtotal resection	493 (3.8%)	372 (4.1%)	10 (1.1%)	111 (3.8%)
Unilateral subtotal resection	219 (1.7%)	151 (1.7%)	2 (0.2%)	66 (2.3%)
Bilateral subtotal resection	125 (0.9%)	86 (0.9%)	7 (0.8%)	32 (1.1%)
Other resections (e.g, isthmus resection, node excision)	428 (3.3%)	167 (1.8%)	5 (0.5%)	256 (8.9%)
All procedures	12,888 (100%)	9069 (100%)	925 (100%)	2894 (100%)

* includes, besides others, follicular adenoma, all forms of thyroiditis, c-cell hyperplasia, normal thyroid; ^#^ *p* < 0.05 Graves’ disease compared to multinodular goitre and other entities combined.

**Table 4:** Intraoperative neuromonitoring, visualisation and autotransplantation of parathyroids.

Procedure	Total (*n* = 12,888)	Multinodular Goitre (*n* = 9069)	Graves’ Disease (*n* = 925)	Other Entities (*n* = 2894)
Neuromonitoring RLN				
no	202 (1.6%)	133 (1.5%)	13 (1.4%)	56 (1.9%)
yes	12,686 (98.4%)	8936 (98.5%)	912 (98.6%)	2837 (98.1%)
intermittent IONM	10,488 (82.6%)	7430 (83.1%)	705 (77.3%)	2352 (82.9%)
continuous IONM	2198 (17.4%)	1506 (16.9%)	207 (22.7%)	485 (17.1%)
Neuromonitoring SLN				
yes	997 (7.7%)	623 (6.9%)	67 (7.2%)	307 (10.6%)
no	11,891 (92.3%)	8446 (93.1%)	858 (92.8%)	2587 (89.4%)
Identification PG				
no	324 (2.5%)	226 (2.5%)	6 (0.6%)	92 (3.2%)
yes	12,564 (97.5)	8843 (97.5%)	919 (99.4%)	2802 (97.8%)
1 PG	1043 (8.1%)	679 (7.5%)	12 (1.3%)	352 (12.2%)
2 or more PG	11,521 (89.3%)	8164 (90%)	907 (98.1%)	2450 (84.7%)
Autotransplantation PG				
yes	1875 (15.3%)	1326 (14.6%)	206 (22.3%) *	343 (11.9%)
no	11,013 (84.7%)	7743 (85.4%)	719 (77.7%)	2551 (88.1%)

RLN—recurrent laryngeal nerve; SLN-external branch superior laryngeal nerve; PG—parathyroid gland; IONM—intraoperative neuromonitoring; * *p* < 0.05 Graves’ disease vs. multinodular goitre and other entities combined.

**Table 5:** Postoperative complications.

Parameter	All Patients (*n* = 12,888)	Multinodular Goitre (*n* = 9069)	Graves’ Disease (*n* = 925)	Other Entities (*n* = 2894)
Function RLN at discharge *				
nerves at risk (NAR)	18,793 (100%)	13,498 (100%)	1766 (100%)	3529 (100%)
not assessed	833 (4.4%)	580 (4.3%)	48 (2.7%)	205 (5.8%)
normal referring to NAR	17,288 (92.0%)	12,424 (92.0%)	1649 (93.4%)	3215 (91.1%)
dysfunction referring to NAR	672 (3.6%)	494 (3.7%)	69 (3.9%)	109 (3.7%)
unilateral paralysis	418 (2.2%)	299 (2.2%)	50 (2.8%)	69 (2.3%)
unilateral incomplete palsy	240 (1.3%)	185 (1.4%)	15 (0.9%)	40 (1.4%)
bilateral paralysis	4 (0.02%)	4 (0.03%)	0	0
bilateral incomplete palsy	10 (0.05%)	6 (0.05%)	4 (0.2%)	0
Hypocalcemia with Ca substitution at discharge ^#^				
yes	1965 (15.3%)	1360 (15%)	263 (28.4%) ^$^	342 (11.9%)
no	10,923 (84.7%)	7709 (85%)	662 (71.6%)	2552 (88.1%)
Reop. for bleeding	180 (1.4%)	104 (1.4%)	12 (1.7%)	20 (1.4%)
Reop. for wound infection	10 (0.07%)	5 (0.6%)	1 (0.1%)	0
Reop. for other reasons (e.g., lymphatic fistula)	6 (0.04%)	5 (0.06%)	0	1 (0.07%)

* vocal cord immobility was defined as paralysis; impaired but notable vocal cord movement was defined as incomplete palsy. RLN—recurrent laryngeal nerve; Ca—calcium; Reop—reoperation; ^#^ postoperative hypoparathyroidism was defined at intact PTH <15 pg/L and serum calcium at <2.1 mmol/L. ^$^ *p* < 0.05 Graves’ disease compared to multinodular goitre and other entities combined.
